# A U-shaped relationship between sleep duration and tinnitus incidence: analysis of 13,871 participants from NHANES

**DOI:** 10.1590/1414-431X2025e14109

**Published:** 2025-03-03

**Authors:** Yongpeng Li, Lu Peng, Ying Lan, Tao Hou, Xiao Pan, Shihua Yin

**Affiliations:** 1 Department of Otorhinolaryngology - Head & Neck Surgery, The Second Affiliated Hospital of Guangxi Medical University, Nanning, China; 2 Department of Otorhinolaryngology - Head & Neck Surgery, Liuzhou People's Hospital Affiliated to Guangxi Medical University, Liuzhou, China; 3 Department of Otorhinolaryngology - Head & Neck Surgery, The Third Affiliated Hospital of Guangxi Medical University, Nanning, China

**Keywords:** Sleep duration, Tinnitus, NHANES, Incidence, U-shaped relationship

## Abstract

Sleep duration is associated to various health impairments, while its comprehensive association with tinnitus is rarely investigated. The current study aimed to explore the relationship between sleep duration and tinnitus incidence, and to determine the optimal sleep duration relating to the lowest tinnitus risk. Data of participants from the National Health and Nutrition Examination Survey (NHANES) from 2005 to 2012 and 2015 to 2018 were retrieved. A total of 13,871 participants were eligible and included in the analysis. Generally, sleep duration was lower in participants with tinnitus compared to those without (7.15±1.76 *vs* 7.30±1.51 h, P<0.001). After adjustment by demographics, lifestyle, and chronic diseases, a U-shaped relationship between sleep duration and tinnitus incidence was observed, with the inflection point at 8.5 h. Interestingly, in participants with sleep duration <8.5 h, sleep duration exhibited an independent negative correlation with tinnitus risk [OR=0.88 (95%CI: 0.84-0.93), P<0.001], while in participants with sleep duration ≥8.5 h, sleep duration had an independent positive association with tinnitus risk [OR=1.16 (95%CI: 1.04-1.28), P=0.006]. In conclusion, a U-shaped relationship was found between sleep duration and tinnitus incidence, with a sleep duration of about 8.5 h being associated with the lowest tinnitus risk.

## Introduction

Tinnitus is a subjective disease mainly caused by noise trauma, metabolic diseases, or ear diseases (1) and affects nearly 15% of the adult population, with an increasing incidence with age (2,3). Tinnitus has put a considerable strain on healthcare systems, patients, their families, and society as a whole, causing heavy financial burden (4,5). Furthermore, 16% of tinnitus cases are categorized as severe, which can lead to insomnia, poor concentration, and psychological distress, significantly reducing the patients' quality of life (3,6,7). Sleep is a vital physiological process that is crucial to human health and performance, whose recommended duration for adults is typically between 7 and 9 h (8). Globally, it appears that people are getting less sleep time on average (9,10). Impaired sleep can produce cognitive and behavioral changes (11); moreover, consistently impaired sleep has been one of the critical risk factors for the development of various disease (12,13). Excessive sleep also causes health problems such as higher risk of cognitive impairment and all-cause mortality (14,15). Interestingly, studies have discovered a U-shaped relationship between sleep duration and various health impairments, including metabolic syndrome, blood pressure, and mental health (16-19).

However, the correlation between sleep duration and tinnitus is rarely comprehensively analyzed. Therefore, the current study retrieved data of 13,871 eligible participants from the National Health and Nutrition Examination Survey (NHANES) from 2005 to 2012 and 2015 to 2018, aiming to confirm the U-shaped association between sleep duration and tinnitus risk, and to determine the inflection point for clinical reference.

## Material and Methods

### Participants

The present study retrospectively analyzed the participants from the NHANES from 2005 to 2012 and 2015 to 2018. The screening criteria were: 1) aged at least 18 years; 2) information of sleep duration was available; 3) information of self-reported tinnitus was available.

### Data retrieval

Sleep duration data were retrieved from the query “How much sleep do you usually get at night on weekdays or workdays?”, which was coded in hours. The occurrence of tinnitus was defined as a positive answer to the query “In the past 12 months, have you been bothered by ringing, roaring, or buzzing in your ears or head that lasts for 5 min or more?”.

Data regarding age, gender, body mass index (BMI), race/ethnicity, education level, marital status, alcohol drinking, cigarette smoking, hypertension, high cholesterol, diabetes mellitus, and hearing loss were also retrieved. Responses such as “Refused” or “Don't know” to any question were recoded as missing data. The percentage of missing data for covariates differed among variables, with diabetes mellitus having the lowest rate at 0.05% and alcohol drinking having the highest rate at 22.4%.

### Statistics

Data are reported as means±SD for continuous factors, and as number and percentage for categorical factors. Comparison was made using the *t*-test or chi-squared test. The univariate and multivariate logistic regression were used to analyze the correlation between sleep duration and tinnitus. Generalized additive model was applied to investigate how sleep duration was related to the occurrence of tinnitus, accounting for covariates. An iterative method was used to determine the sleep duration threshold by selecting the inflection point within a predefined range that led to the maximum likelihood model. Sensitivity analyses were made by excluding individuals with sleep durations <4 or >12 h to test whether the poor health of participants confounded the results. R project (R Foundation for Statistical Computing, Austria) and EmpowerStats (X & Y Solutions, Inc., USA) softwares were used. A P-value below 0.05 was defined as significant.

## Results

### Study flow

**Figure 1:**
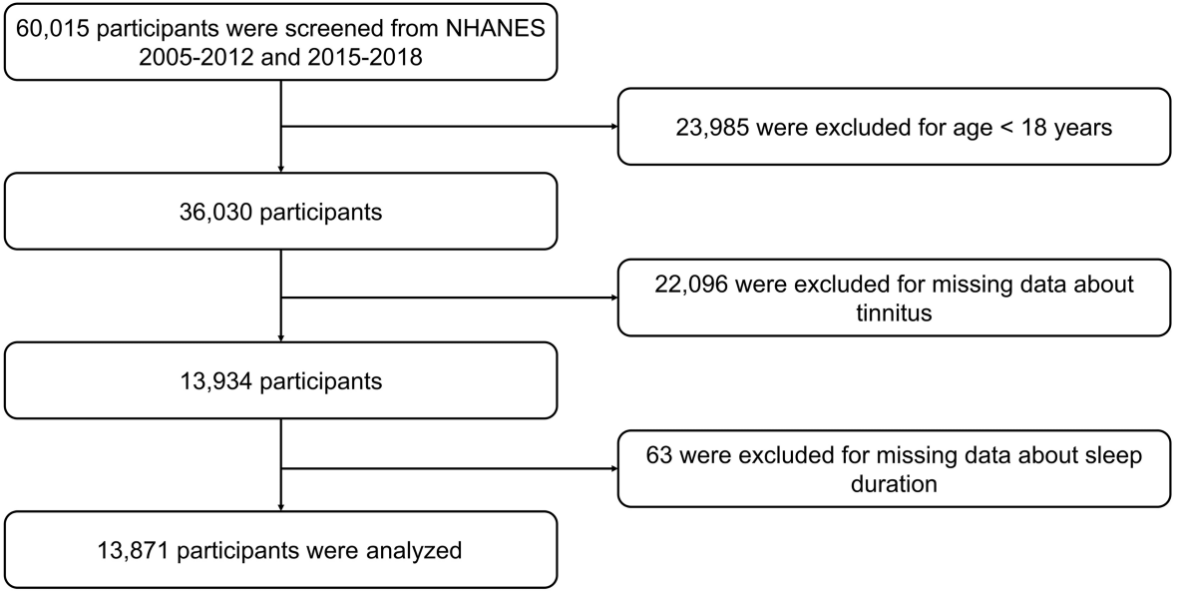
Study flow chart.

Initially, 60,015 participants were screened from NHANES 2005-2012 and 2015-2018; then, 23,985 cases were excluded for age <18 years, 22,096 cases were excluded for missing data about tinnitus, and 63 cases were excluded for missing data about sleep duration. Finally, 13,871 participants were analyzed in the current study (Figure 1).

### Characteristics of participants

The mean age of total participants was 48.4±20.6 years, and the sample included 49% males and 51% females (Table 1). Of total participants, 2,169 (16%) cases had tinnitus, while 11,702 (84%) did not. By comparison, it was observed that age (P<0.001), gender (P=0.026), BMI (P<0.001), race/ethnicity (P<0.001), education level (P<0.001), marital status (P<0.001), alcohol drinking (P<0.001), hypertension (P<0.001), diabetes (P<0.001), and hearing loss (P<0.001) were different between participants with tinnitus and those without.

**Table 1:** Patients' characteristics.

Characteristics	Total participants (n=13,871)	Tinnitus		P value
		Yes (n=2,169)	No (n=11,702)	
Age, mean±SD (years)	48.4±20.6	55.7±19.4	47.0±20.6	<0.001
Gender, n (%)				0.026
Male	6806 (49%)	1112 (51%)	5694 (49%)	
Female	7065 (51%)	1057 (49%)	6008 (51%)	
BMI, mean±SD (kg/m^2^)	28.7±7.0	30.1±7.1	28.5±6.9	<0.001
Race/ethnicity, n (%)				<0.001
Mexican American	2015 (15%)	330 (15%)	1685 (14%)	
Other Hispanic	1417 (10%)	206 (9%)	1211 (10%)	
Non-Hispanic White	5299 (38%)	1062 (49%)	4237 (36%)	
Non-Hispanic Black	3178 (23%)	386 (18%)	2792 (24%)	
Other race	1962 (14%)	185 (9%)	1777 (15%)	
Education level, n (%)				<0.001
Less than 9th grade	1342 (11%)	260 (13%)	1082 (10%)	
9th to 11th grade	1685 (14%)	319 (16%)	1366 (13%)	
High school graduate	2734 (22%)	484 (24%)	2250 (22%)	
Some college or AA degree	3626 (29%)	614 (30%)	3012 (29%)	
College graduate or above	3007 (24%)	349 (17%)	2658 (26%)	
Marital status, n (%)				<0.001
Married	6205 (48%)	979 (47%)	5226 (48%)	
Widowed	1300 (10%)	275 (13%)	1025 (9%)	
Divorced	1253 (10%)	274 (13%)	979 (9%)	
Separated	409 (3%)	81 (4%)	328 (3%)	
Never married	2763 (21%)	326 (16%)	2437 (22%)	
Living with partner	1049 (8%)	141 (7%)	908 (8%)	
Alcohol drinking, n (%)				<0.001
Yes	7723 (72%)	1381 (75%)	6342 (71%)	
No	3037 (28%)	457 (25%)	2580 (29%)	
Cigarette smoking, n (%)				<0.001
Yes	5372 (42%)	1061 (52%)	4311 (41%)	
No	7309 (58%)	995 (48%)	6314 (59%)	
Hypertension, n (%)				<0.001
Yes	4850 (35%)	1062 (49%)	3788 (32%)	
No	9000 (65%)	1103 (51%)	7897 (68%)	
High cholesterol, n (%)				<0.001
Yes	4288 (35%)	944 (47%)	3344 (32%)	
No	8091 (65%)	1051 (53%)	7040 (68%)	
Diabetes mellitus, n (%)				<0.001
Yes	1764 (13%)	378 (17%)	1386 (12%)	
No	11805 (85%)	1723 (79%)	10082 (86%)	
Borderline	295 (2%)	67 (3%)	228 (2%)	
Hearing loss, n (%)				<0.001
Yes	4931 (42%)	1217 (64%)	3714 (37%)	
No	6867 (58%)	671 (36%)	6196 (63%)	

SD: standard deviation; BMI: body mass index. Comparisons were made using the *t*-test or chi-squared test.

### Generalized analyses between sleep duration and tinnitus

**Figure 2:**
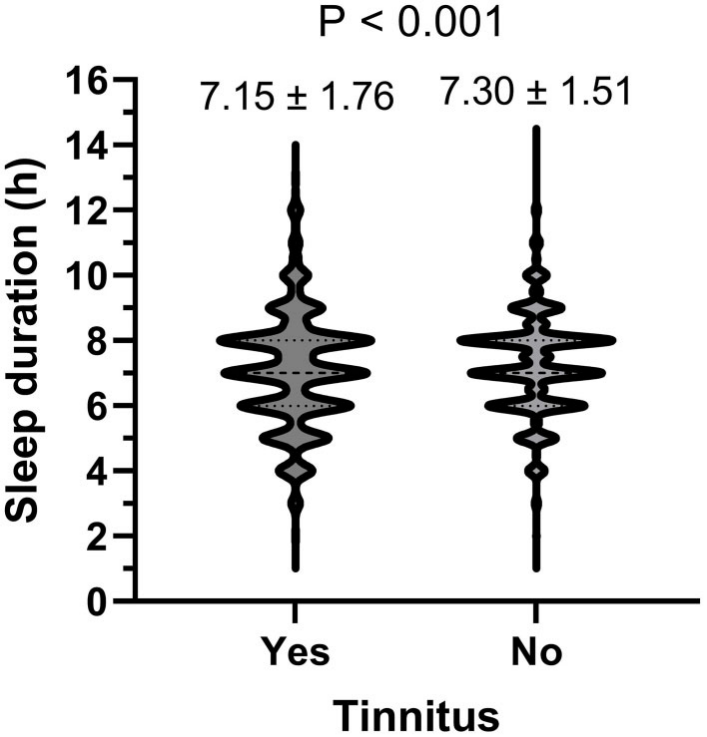
Comparison of sleep duration in tinnitus participants and non-tinnitus participants. The mean sleep duration of each group in indicated. The Student’s *t*-test was used for analysis.

Generally, sleep duration was lower in participants with tinnitus compared to those without (7.15±1.76 *vs* 7.30±1.51 h, P<0.001) (Figure 2). The violin graph showed that participants with tinnitus more frequently had excessive or sufficient sleep distribution than those without tinnitus.

Subsequently, logistic regression analysis showed that regardless of the model, sleep duration was negatively associated with tinnitus risk (Table 2). The models were as follows: Model 1: unadjusted; Model 2: adjusted by age, gender, and race/ethnicity; Model 3: adjusted by Model 2 + education level, marital status, body mass index, cigarette smoking, alcohol drinking, diabetes mellitus, hypertension, high cholesterol, and hearing loss.

**Table 2:** Correlation between sleep duration and tinnitus risk by multivariate logistic regression.

Parameter	Model 1		Model 2		Model 3	
	OR (95%CI)	P value	OR (95%CI)	P value	OR (95%CI)	P value
Sleep duration	0.94 (0.91-0.97)	<0.001	0.92 (0.90-0.95)	<0.001	0.94 (0.91-0.97)	<0.001

Model 1: unadjusted. Model 2: adjusted by age, gender, and race/ethnicity. Model 3: adjusted by Model 2 + education level, marital status, body mass index, cigarette smoking, alcohol drinking, diabetes mellitus, hypertension, high cholesterol, and hearing loss. OR: odds ratio; CI: confidence interval.

### U-shaped relationship between sleep duration and tinnitus incidence

**Figure 3:**
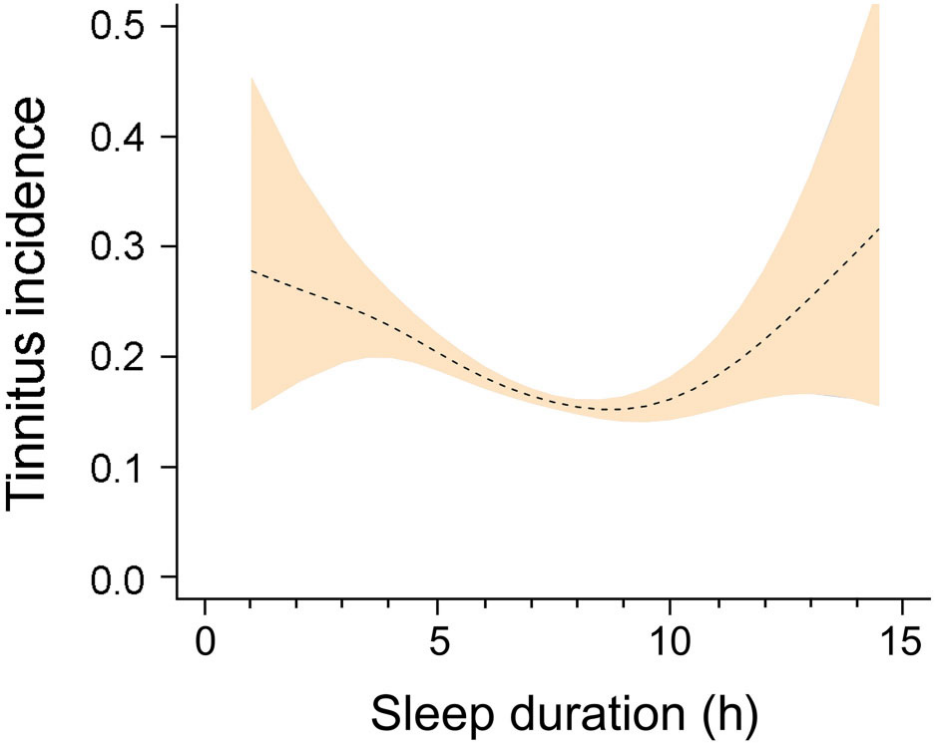
U-shaped relationship between sleep duration and tinnitus incidence in total participants. The peach color indicates 95%CI of tinnitus incidence.

Based on the different associations between sleep duration and tinnitus risk in subgroups of participants with varied sleep duration stratifications, it was considered that their relationship might be not linear. The additive method was then applied, which revealed a U-shaped relationship between sleep duration and tinnitus incidence (Figure 3), with the inflection point at 8.5 h.

Thereafter, participants were stratified based on the sleep duration of 8.5 h, and in participants with sleep duration <8.5 h. Sleep duration of 8.5 h exhibited an independent negative correlation with tinnitus risk, while in participants with sleep duration ≥8.5 h, sleep duration revealed an independent positive association with tinnitus risk (Table 3).

**Table 3:** Correlation between sleep duration and tinnitus risk in subgroups divided by the inflection point of 8.5 h.

Parameter	OR (95%CI)	P value
Participants with sleep duration <8.5 h (n=11,221)	0.88 (0.84-0.93)	<0.001
Participants with sleep duration ≥8.5 h (n=2,650)	1.16 (1.04-1.28)	0.006

Adjusted by age, gender, race/ethnicity, education level, marital status, body mass index, cigarette smoking, alcohol drinking, diabetes mellitus, hypertension, high cholesterol, and hearing loss. OR: odds ratio; CI: confidence interval.

### Sensitivity analyses

**Figure 4:**
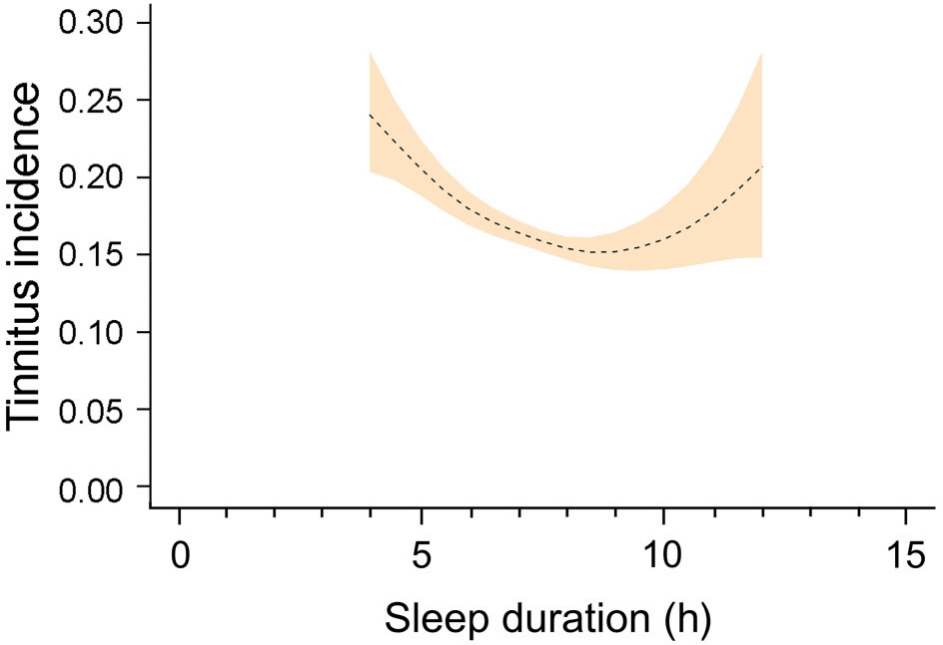
U-shaped relationship between sleep duration and tinnitus incidence in participants with sleep duration between 4 and 12 h. The peach color indicates 95%CI of tinnitus incidence.

To check the stability of the U-shape relationship found between sleep duration and tinnitus incidence, participants with extreme sleep duration values (defined as: <4 or >12 h) were excluded from the analysis, and the U-shape relationship was maintained (Figure 4). Furthermore, in participants with sleep duration 4-8.5 h, sleep duration was negatively associated to tinnitus risk, and in participants with sleep duration 8.5-12 h, sleep duration was positively associated with tinnitus risk (Table 4).

**Table 4:** Correlation between sleep duration (4-12 h) and tinnitus risk in subgroups divided by inflection point 8.5 h.

Parameter	OR (95%CI)	P value
Participants with sleep duration 4-8.5 h (n=11,081)	0.88 (0.84-0.92)	<0.001
Participants with sleep duration 8.5-12 h (n=2,619)	1.14 (1.02-1.28)	0.027

Adjusted by age, gender, race/ethnicity, education level, marital status, body mass index, cigarette smoking, alcohol drinking, diabetes mellitus, hypertension, high cholesterol, and hearing loss. OR: odds ratio; CI: confidence interval.

## Discussion

The current study observed a U-shaped relationship between sleep duration and tinnitus, with a sleep duration of approximately 8.5 h associated with the lowest tinnitus risk. Total sleep duration <8.5 h increased the risk of tinnitus onset by 12% for every hour less of sleep. Total sleep duration ≥8.5 h increased the risk of tinnitus by 16% for every additional hour of sleep. These associations were independent of age, gender, race/ethnicity, education level, marital status, cigarette smoking, alcohol drinking, diabetes mellitus, hypertension, high cholesterol, BMI, and hearing loss.

Sleep is closely related with tinnitus. Sleep duration is one of the critical dimensions to define and measure sleep health (20). Studies have revealed a connection between sleep health and the likelihood of suffering tinnitus (21-23). Additionally, insufficient sleep duration can worsen the severity of tinnitus (24-27). Reversely, treating tinnitus leads to improved sleep health, with the level of improvement correlating to the improvement of tinnitus severity (28). Our current study also discovered that inadequate sleep duration was related to a higher likelihood of tinnitus incidence. Although much is known regarding the implications of deficient sleep duration on tinnitus, little attention has been paid to the association between excessive sleep duration and tinnitus. Our study also observed that longer sleep duration can be a considerable risk for tinnitus. However, one study found no association between longer sleep duration and tinnitus incidence (29). This could be because each study considered different potential confounding factors. Furthermore, the age ranges of participants in each study varied, with one enrolling participants aged 49 to 69 years in the United Kingdom and one enrolling participants aged 18 to 85 years in the NHANES database.

The mechanism underlying the interaction between deviated sleep duration and tinnitus has yet to be discovered. Some proposed mechanisms explain how impaired sleep can lead to the development of tinnitus. First, insomnia and tinnitus share similar pathophysiology. Insomnia patients with tinnitus have no difference in sustained attention abilities compared to those without tinnitus (21). Further studies revealed notable similarities in the activation patterns of limbic and autonomous brain regions between insomnia and tinnitus (30,31). Second, impaired sleep may cause tinnitus mediated by hearing loss. As hearing loss is one of the significant risk factors for tinnitus, tinnitus is often deemed a neuroplastic phantom response to hearing impairment (32-34). In addition, studies have revealed that insufficient or excessive sleep duration is closely associated with hearing loss (35-37).

Several limitations could be pointed out when interpreting the findings of this study. Firstly, the cross-sectional design of NHANES does not allow for causal conclusions. Future longitudinal and intervention studies will help fully understand the impact of sleep duration on the risk of developing tinnitus. Second, all the reports of tinnitus and sleep duration were based on subjective data, possibly leading to reporting biases. Therefore, it is recommended to conduct longitudinal assessments of both objective and subjective sleep duration. Finally, the study considered several retrievable covariates that could impact the findings, but there is still a chance that some variables, whether measured or unmeasured, may influence the results.

In conclusion, a U-shaped relationship was found between sleep duration and tinnitus incidence, in which a sleep duration of approximately 8.5 h was associated with the lowest tinnitus risk.
